# Assessment of the accuracy of a new tool for the screening of smartphone addiction

**DOI:** 10.1371/journal.pone.0176924

**Published:** 2017-05-17

**Authors:** Julia Machado Khoury, André Augusto Corrêa de Freitas, Marco Antônio Valente Roque, Maicon Rodrigues Albuquerque, Maila de Castro Lourenço das Neves, Frederico Duarte Garcia

**Affiliations:** 1 Department of Mental Health, Universidade Federal de Minas Gerais, Belo Horizonte, Brazil; 2 Post-graduation Program in Molecular Medicine, School of Medicine, Universidade Federal de Minas Gerais, Belo Horizonte, Brazil; 3 Department of Sports, School of Physical Education, Universidade Federal de Minas Gerais, Belo Horizonte, Brazil; 4 National Institute of Science and Technology of Molecular Medicine, Universidade Federal de Minas Gerais, Belo Horizonte, Brazil; 5 Unité Inserm, Rouen, France; Hospital Universitari de Bellvitge, SPAIN

## Abstract

**Objective:**

To translate, adapt and validate the Smartphone Addiction Inventory (SPAI) in a Brazilian population of young adults.

**Method:**

We employed the translation and back-translation method for the adaptation of the Brazilian version SPAI (SPAI-BR). The sample consisted of 415 university students. Data was collected through an electronic questionnaire, which consisted of the SPAI-BR and the Goodman Criteria (gold standard). The retests were carried out 10–15 days after the initial tests with 130 individuals.

**Results:**

The SPAI-BR maintained semantic, idiomatic and conceptual equivalences from the original scale. The Confirmatory Factor Analysis confirmed the One-factor model of the SPAI with good fit indexes (x^2^ = 767.861, CFI = 0.913, TLI = 0.905, RMSE = 0.061, WRMR = 1.465). The Kuder-Richardson Coefficient showed good internal consistency. The analysis of the ROC curve established an area under the curve of 86.38%. The Intraclass-Correlation Coefficient of 0.926 between the test and the retest demonstrated an excellent temporal stability. The high correlation between SPAI-BR and the Goodman Criteria (rs = 0.750) established the convergent validity.

**Conclusion:**

The SPAI-BR is a valid and reliable tool for the detection of Smartphone Addiction in Brazilian university students.

## Introduction

Smartphones are essential tools in our everyday life. They provide applications for communication, information, education, and entertainment. They have also been used for data collection, to prevent and treat psychiatric disorders, chronic diseases and to improve elders’ quality of life [1–9]. Although smartphones can improve many aspects of our lives, excessive use may be associated to smartphone addiction [10–13].

Smartphone addiction is a technological addiction, defined by Griffiths as a behavioral addiction that involves human-machine interaction [14]. Several authors have described the presence of addiction symptoms in subjects presenting a problematic use or diagnosed with smartphone addiction. Among these, the most commonly described were withdrawal symptoms (i.e. anxiety, irritability, and impatience) [11–13, 15–19]; loss of control in using smartphones [15, 20]; a longer time of use than initially intended [10–13]; tolerance [11–13, 19]; interference in activities of daily living [10–13, 19, 21]; positive anticipation [11, 18, 19, 22]; and maintenance of the amount of use despite negative consequences [21].

Studies in several countries reported a high prevalence of smartphone addiction, especially among university students. The prevalence of smartphone addiction in young students is estimated at 6% in Italy [23]; 38% in Spain [16]; 18.8% in Japan[24]; 28.7% in the Netherlands [25]; 27.4% in Hong Kong [26]; 25% in the United States[27]; 44% in India [28]; 25.8% in Jordan [29]; and 67% in the United Arab Emirates [30].

In terms of negative consequences, the diagnosis of smartphone addiction is associated with sleep disorders [31–33]; depressive and anxious symptoms [31, 33–36]; and reduction of academic and labor performance [37, 38].

Although no specific diagnostic criteria for smartphone addiction exist, the study of this disorder seems to be an important issue. Screening instruments are important as they are a first step to the phenotyping process of research. No validated instrument for the screening of smartphone addiction is available in Brazil. Around the globe, the “Smartphone Addiction Scale” (SAS)[11] and, the “Smartphone Addiction Inventory” (SPAI) are the most frequently used screening instruments [39]. We opted to validate the SPAI questionnaire, because it is shorter and easier to respond, therefore, more suitable to be used in the Brazilian public health care system.

The main aim of this study was to validate the SPAI for use in the Brazilian population. We hypothesize that a Brazilian version of the SPAI (SPAI-BR) is a valid tool for the screening of smartphone addiction in Brazilian young adults.

## Materials and methods

### Study design and ethical aspects

This was a cross-sectional and prospective study for the assessment of psychometric features of the Brazilian version of the Smartphone Addiction Inventory. The Committee of Ethics in Research of the Federal University of Minas Gerais (UFMG) approved this study (CAAE 54066516.0.0000.5149). Participants provided their written informed consent about the voluntary nature of the study, its risks, and its benefits. This study did not include minors and it was carried out in accordance with the latest version of the Declaration of Helsinki.

### Setting and sample

This study was developed at the Universidade Federal de Minas Gerais (UFMG) from March to June of 2016. We recruited a convenience sample of students from different graduate courses at UFMG.

All undergraduate students that have a smartphone with all day internet access (e.g. 3G, 4G or Wi-Fi), excluding subjects with visual or hearing impairment, were eligible to participate. We based the sample profile on previously recognized risk factors for smartphone addiction [16, 23, 25–28, 30, 40, 41].

We calculated the sample size based on the recommendations of Hair et al. [42], to use at least 10 times the number of subjects for each item on the scale. As the SPAI has 26 items, the calculated sample for this study was of 450 individuals. The retest sample size calculation was based on the study of correlations of Faraggi and Reiser [43]. According to the recommendation of these authors, the sample size calculation for correlation studies should consider the probability of type I error, the probability of type II error and the hypothetical value of the expected correlation coefficient in the study. For a correlation coefficient of 0.60 (good correlation) and considering the probabilities of occurrence of type I error (0.05) and type II error (0.10), each group should be composed of a minimum of 25 subjects, totaling a minimum of 50 individuals who undergo the test-retest. We estimated a sample size of 150 subjects for retest because the non-response rate in previous studies, assessing retest by e-mail, presented a non-response rate of 60 to 70% [44].

### Instruments

#### Smartphone Addiction Inventory (SPAI)

Lin and Chang developed and validated the SPAI for the screening of Smartphone Addiction in a Taiwanese sample of graduate students in 2014 [39]. Authors based the SPAI on the "Chen Internet Addiction Scale" (CIAS) [45]. In summary, the term “internet” in the CIAS was replaced by “smartphone” and authors added one question to assess the use of smartphone while driving or crossing the street. The original SPAI has 26 items in Likert format. The Cronbach Alpha value of the entire scale is 0.94 and for the four subscales, "compulsive behavior", "functional impairment", "withdrawal syndrome" and "tolerance syndrome" it is 0.87, 0.88, 0.81 and 0.72, respectively.

Pavia et al. (2016) translated, adapted and validated an Italian version of the SPAI in a sample of 485 college students from the University of Palermo in Sicily [46]. Non-validated versions of the SPAI have been used in South Korea and Spain [47, 48].

#### Goodman’s criteria

Due to the lack of a specific diagnostic criteria for smartphone addiction, we used Goodman’s Criteria for addictions as the gold standard for this study [49]. Goodman’s Criteria were used as a basis for the construction of addictive disorders’ diagnostic criteria of ICD-10 [50] and DSM-5 [51] and they have been widely used in an interview format by specialists to diagnose dependency syndromes [49, 52–59]. Previous validation studies of screening instruments for behavioral addictions that did not have specific diagnostic criteria employed Goodman's criteria as the gold standard [52–55, 57, 59].

### Translation and cultural adaptation of the SPAI

The SPAI was translated into Brazilian Portuguese language and edited for syntax and content by five experts on addictions. After adjustments, it was back translated by a native English speaker linguist. Original and back-translated versions were reviewed, compared and adjusted for equivalence by consensus among the study team and the linguist. Additional editing of the Brazilian version of the SPAI (SPAI-BR) was made to improve the understanding of the questions after a pilot study performed on a group of 10 students. Moreover, we opted to use a dichotomic version of the SPAI-BR in order to decrease time of completion and increase efficiency for its use in health surveys. Finally, we performed a second pilot test with 40 graduate students using the final version of the SPAI-BR to assess mean time of questionnaire completion.

### Data collection

The final questionnaire was composed of three sections: 1. demographic data: gender, race, birth date, marital status, and family income; 2. SPAI-BR; and 3. Goodman’s Criteria. For data collection, we used an electronic data collection system based on tablet computers. In sum, we transferred the whole questionnaire to the web-based platform iSurvey® (HarvestYourData, CA) and installed the software on tablet computers. Respondents completed the demographic questionnaire and the SPAI-BR (self-applied) and handed the tablet to a psychiatrist who performed a structured interview assessing Goodman’s Criteria. Data was uploaded through Wi-Fi, transmitted to a database and exported to SPSS format (IBM Corporation, CA). Data bank in SPSS format can be seen in S1 File.

### Statistical analysis

In the descriptive analysis, we calculated mean, standard deviation, median, quartiles and minimum /maximum for continuous variables. For categorical variables, we calculated frequency and proportion. Data normality was assessed using Kolmogorov-Smirnov test.

Factorial structure of the SPAI-BR was evaluated using Confirmatory Factor Analysis (CFA). We used the Weighted Least Squares Mean and Variance estimator (WLSMV) as a method for parameter estimation. Model fit was assessed considering the following fit indexes: Chi-square (χ ^2^), Comparative Fit Index (CFI), Tucker-Lewis Index (TLI), Root Mean Square Error (RMSE) and Weighted Root Mean Square Residual (WRMR). The overall model fit was judged using the following cutoff values: for the CFI and TLI, values larger than 0.90 are considered indicators of acceptable fit [60–62]; for the RMSE, values smaller than 0.05 indicate good fit and values between 0.05 and 0.08 indicate acceptable model fit [63]; for the WRMR, a cutoff value close to 1.00 is considered suitable [64]. Preliminarily Kaiser-Meyer-Olkin (KMO) and Bartlett Test of Sphericity were used to examine the factorability of the data. Internal consistency was computed using Kuder-Richardson Coefficient for the total scale and for the dimensions extracted by factor analysis.

To calculate correlation between the scores of the SPAI-BR at different times (test-retest) we used the Intraclass-Correlation Coefficient, which indicates temporal stability of the instrument. Correlations were evaluated according to the following values: less than 0.40 –poor correlation; 0.41 to 0.60 –moderate correlation; 0.61 to 0.80 –good correlation; and 0.81 to 1.00 –excellent correlation [65].

Several "Receiver Operating Characteristic" (ROC) curves were constructed to evaluate the SPAI-BR and its sensitivity, specificity, positive predictive value and negative predictive value at different levels of prevalence.

Additionally, to derive optimal cut-off scores, decision theory was used as Smits and colleagues have proposed for mental health screening [66, 67]. Medical costs were included in our analyses of relative costs considering that expenses in a mental health setting will be similar for patients with smartphone addiction (67). Using the proportional costs of correct and erroneous screening results and the rates of these correct and erroneous screening results according to prevalence, we calculated relative costs for each cut-off point, with the formula:

$$cost=(C_{TP}\times P_{TP})+(C_{TN}\times P_{TN})+(C_{FP}\times P_{FP})+(C_{FN}\times P_{FN})$$

In this formula, “cost” represents total medical costs, C_TP_ stands for the costs of each true positive, and P_TP_ represents the probability of true positives (according to the levels of prevalence, specificity and sensibility). Subsequently, costs and probabilities are analyzed for true negatives (TN), false positives (FP) and FN (false negatives). We calculated relative costs with the proportions of expenses for true positives (0.89), true negatives (0.001), false positives (0.03) and false negatives (1.0) as described by Smits and colleagues [66, 67].

As proposed by decision theory, the optimal cut-off point was chosen according to lowest expected costs.

To determine the convergent validity between SPAI-BR and Goodman’s Criteria we calculated the Spearman Correlation Coefficient for the total scale and the factors.

In this study, each subject responded a questionnaire on an electronic platform, and the non-response of an item precluded the progression to the next items. For this reason, our database did not include missing values.

Analyses were performed in STATA 12.1 software (Stata Corporation, College Station, Texas) and MPLUS version 6.12.

## Results

### Translation and cultural adaptation

The translation and cultural adaptation led to an instrument in Brazilian Portuguese that kept the semantic (meaning of words), idiomatic (meaning of expressions) and conceptual (meaning of concepts) equivalences from the original scale. The original scale and the translated scale are represented in S1 Table.

### Sample description

At endpoint, we assessed 415 subjects, and retested 130 individuals at 10 to 15 days (Mean _=_ 12.619 ± 1.593) after the initial test. The demographic characteristics of the sample are described in Table 1. In the entire sample, the prevalence of smartphone addiction is 35.66% (n = 148) according to Goodman’s Criteria. The flow diagram and the cross tabulation of the index test results by the results of the gold standard can be seen in S1 Fig.

**Table 1:** Sample demographic characteristics (n = 415).

Parameter		n	%
Gender	Female	226	54.5
	Male	189	45.5
Age	18 to 25	321	77.3
	26 to 35	94	22.7
Marital status	Married	21	5.1
	Unmarried	394	94.9
Skin color	Indian	10	2.4
	White	252	60.7
	Black	19	4.6
	Brown	116	28.0
	Did not answer	18	4.3
Monthly family income	No income	7	1.7
	Up to R$880,00	10	2.4
	From R$880,00 to R$ 2.640,00	55	13.3
	From R$2.640,00 to R$5.280,00	71	17.1
	From R$5.280,00 to R$7.920,00	65	15.7
	From R$7.920,00 to R$10.560,00	52	12.5
	From R$10.560,00 to R$13.200,00	34	8.2
	From R$13.200,00 to R$17.600,00	38	9.2
	Above R$ 17.600,00	43	10.4
	Do not know/did not answer	40	9.6

### Confirmatory factor analysis

**Fig 1:**
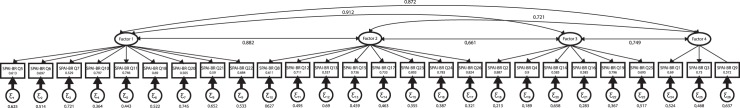
Confirmatory factor analysis’s path diagram. F1: ‘Compulsive Behavior’; F2: ‘Functional Impairment’; F3: ‘Withdrawal’; F4: ‘Tolerance’; SPAI 1–26, correspond to each of the SPAI-BR questions, respectively.

The goodness of fit, assessed by the KMO, was of 0.901, and the Bartlett’s Sphericity Test resulted in 2964.30 (p <0.001), considering the 26 questions. The responses of the SPAI-BR were not normally distributed according to the Kolmogorov-Smirnov Test (p <0.001), therefore, we used the method of weighted least squares and adjusted variance (WLSMV) for the Confirmatory Factor Analysis (CFA). Moreover, for the CFA we considered four factors with oblique rotation, as previously described in the original validation study of the SPAI. Both models resulted in a good fit index, as can be seen in Table 2 and in the CFA path diagram in Fig 1.

**Table 2:** Fit Indices for the confirmatory factor analysis models (n = 415).

	Χ^2^	df	CFI	TLI	RMSE	WRMR
One- Factor	767.861	299	0.913	0.905	0.061 (0.056–0.067)	1.465
Four—Factors (oblique)	626.482	293	0.938	0.931	0.052 (0.047–0.058)	1.289

Notes: CFI = Comparative Fit Index; df = degrees of freedom; RMSE = Root Mean Square Error; TLI = Tucker-Lewis Index; WRMR = Weighted Root Mean Square Residual; χ ^2^ = chi-square.

### Internal consistency

The Kuder-Richardson Coefficient of the One-factor model was 0.887. Moreover, the Kuder-Richardson Coefficient of the Four-factor model was 0.738 for ‘Compulsive Behavior’; 0.736 for ‘Functional Impairment’; 0.753 for ‘Withdrawal’; and 0.481 for ‘Tolerance’. Since the ‘Tolerance’ factor has shown low internal consistency (less than 0.7), the One-factor structure is more adequate for SPAI-BR.

### Temporal stability

We retested 130 subjects after 10 to 15 days (Mean _=_ 12.619 ± 1.593) from the initial test. The Intraclass-Correlation Coefficient (ICC) was 0.926, indicating an excellent positive correlation between test and retest (F = 27.55, p < 0.01). The temporal stability of the SPAI-BR is presented in S2 Table.

### Criterion validity

Criterion validity was established through the comparison between SPAI-BR and the Goodman Criteria (the gold standard used in the study). We ran ROC curves considering the prevalence of smartphone addiction in our study (35.66%) and considering other approximate prevalences that have been found in the literature (5%; 10%, 20% and 40%) (S3 Table). The ROC curve for the prevalence found in our study (S2 Fig.) has an area under the curve of 86.38% (standard error 0.0183), meaning that the SPAI-BR has good accuracy in our population[68]. In general, higher AUC values indicate better test performance [69].

At the prevalence found in our study (35.66%), the optimal cut-off point with the lowest costs was seven. At this cut-off, sensitivity was 90.54%, specificity was 59.93%, PPV was 55.6, NPV was 92.0% and accuracy was 70.85%. Additional cost estimates relative to different prevalences found in the literature are displayed in S4 Table.

### Convergent validity

In the convergent validity analysis, the correlations between the SPAI-BR score and Goodman Criteria score were examined. The SPAI-BR total score was found to have a positive correlation with the Goodman Criteria total score at Spearman's rho of 0.751. The correlations between the SPAI-BR subscales and the Goodman Criteria total score ranged from 0.548 to 0.709, indicating a statistically significant positive correlation (p < 0.01). Table 3 shows the values of convergent validity.

**Table 3:** Spearman Correlation Coefficients between the total SPAI-BR, the SPAI-BR’s factors and the Goodman Criteria (n = 415).

	Goodman	Factor 1	Factor 2	Factor 3	Factor 4	SpaiTotal
Goodman	1					
Factor 1	0.7093	1				
Factor 2	0.5528	0.6125	1			
Factor 3	0.6264	0.6924	0.4969	1		
Factor 4	0.5481	0.5705	0.4467	0.5225	1	
SpaiTotal	0.7508	0.9047	0.7699	0.8504	0.7078	1

Note: Factor1: “Compulsive Behavior”, Factor 2: “Functional Impairment”, Factor 3: “Withdrawal”, Factor 4: “Tolerance”. All correlations are significant for p < 0.001.

## Discussion

The purpose of this study was to translate the English version of an instrument for smartphone addiction screening, the SPAI, into Brazilian Portuguese and achieve cultural adaptation and validation of this Brazilian version (SPAI-BR). Our results demonstrated a good validity and reliability of the SPAI-BR for the detection of Smartphone addiction in a Brazilian population of young adults.

In this study, we validated a dichotomic (i.e. “yes”; “no”) version of the questionnaire, aiming for reduction in completion time and making the SPAI-BR more suitable for the Brazilian population. This procedure has been questioned in the literature since dichotomic format may decrease the instrument's sensitivity and its internal consistency [70]. However, some studies have shown that dichotomous format is of more rapid application and easier to understand [44, 70, 71]. Therefore, this format seems to be more appropriate for populations with low socio-economic-cultural level, as is characteristic in much of the Brazilian population. Furthermore, our results demonstrated that the SPAI-BR provided an internal consistency and a sensitivity comparable to the original version of the questionnaire, even in a dichotomic version [39].

We performed the Confirmatory Factor Analysis (CFA) in our dataset to assess if our instrument presented a factorial structure similar to the original SPAI [72, 73]. CFA showed good model fit indexes for both the one-factor model and the Four-factor model. However, the ‘Tolerance’ factor presented a lower index of internal consistency (less than 0.7). The adequacy of the presence of tolerance for the diagnosis and for the screening of smartphone addiction has been questioned. It is controversial whether tolerance is a core symptom of smartphone addiction. "Tolerance" is the factor with the most unstable structure, the smallest eigenvalue, and the fewest number of items in most smartphone addiction questionnaires [11, 39, 74]. Additionally, tolerance in smartphone addiction is difficult to measure, since smartphone use is carried out for short periods of time, with great frequencies, and the total time of use is very inaccurately self-reported. Smartphones have also become essential to current lifestyles. Therefore, increased smartphone use may not be pathological, but rather a necessity for functional communication.

Besides all the reasons above, we attribute the low internal consistency of the “tolerance” factor to the fact that we have converted the original Likert instrument into a dichotomous format and because the ‘Tolerance’ factor is formed by only three items. Internal consistency is normally influenced by both factors, as demonstrated in previous studies [72, 73]. Kline [75] suggests that values lower than 0.7 are expected and acceptable in psychological constructs because of the diversity of the structures being measured. Moreover, Streiner (2003) estimates that, although an internal consistency coefficient of 0.40 may be considered insufficient for diagnostic tests, it is acceptable for screening tests. We opted to consider the one-factor SPAI-BR more appropriate for screening, as there is no consensus regarding the suitability of the internal consistency of less than 0.7 in these cases. Furthermore, the one-factor structure demonstrated optimal internal consistency (KR = 0.887) and good model fit indexes [χ^2^ = 767.861; CFI = 0.913, TLI = 0.905, RMSE = 0.061; WRMR = 1.465], and it does not change the clinical applicability of the instrument.

The temporal stability of the SPAI-BR was excellent (ICC = 0.926), which shows the reliability of the instrument. In addition, the positive correlation between the SPAI-BR and the Goodman Criteria (Spearman's rho = 0.751) indicated that both instruments allow the detection of the same construct, establishing the convergent validity of the SPAI-BR.

Regarding the determination of possible cutoff points for SPAI-BR, some considerations must be made. The choice of a cutoff point will depend on the purpose of the instrument (diagnosis or screening) and the prevalence of smartphone addiction in the population in which the test will be applied. In addition, no cutoff point has full accuracy, so diagnostic errors will always occur.

The decision theory approach considers costs and benefits of correct and incorrect classifications through the screening instrument [66, 67]. Some authors have attempted to analyze these costs from a series of perspectives such as a respondent perspective, a health service provider perspective, a societal perspective and a research perspective [66, 67, 76, 77]. Economically speaking, the health care provider and the societal perspective can prove more interesting. However, since there is no previous reference to the costs of smartphone addiction, for the purposes of cost analyses, we limited ourselves to the medical costs of smartphone addiction, assuming that these can be generalized in a mental health setting (67).

We must consider that both correct and incorrect classifications by a screening instrument will incur in medical costs. Lower cutoff points cause more false positives, increasing direct treatment costs. Higher cutoff points generate more false negatives, harming many ill individuals who will not be treated. In this sense, we used the same proportions of costs of screening and treatment in a mental health setting as established by Smits and colleagues [66, 67]. In our population, with prevalence of 35.66%, the lowest costs are found at a cut-off point of seven. For this optimal cutoff point, sensitivity was 90.54%, much greater than specificity (59.93%), and, although specificity can be considered low, this is adequate for a screening instrument [43]. Additionally, the cut-off point of seven has a high accuracy (70.85%) in our population. Investigators and clinicians may consider a distinct cut-off point according to the expected prevalence and estimated costs in their target population, as displayed in S4 Table.

One possible limitation of this study was the fact that it was carried out with a convenience sample composed of UFMG students, and therefore it could be expected that the application of this same instrument in a community sample would present different results. Therefore, data from this study cannot be generalized to other populations. However, the instrument validation process is continuous and, generally, its initial phase is carried out with the population at greater risk for the construct under analysis. Future studies with heterogeneous populations are required so that SPAI-BR will be considered valid for application across the general population.

Another limitation concerns the gold standard used to diagnose smartphone addiction in this study. The lack of a validated gold standard for the screening of smartphone addiction may compromise the accuracy of criterion validity of the SPAI-BR. However, Goodman’s Criteria is the best approximation of a gold standard, as is very similar to the diagnostic criteria for addictions in DSM 5 and ICD 10.

In conclusion, present data confirms that SPAI-BR is accurate and reliable for the quick and easy detection of patients with smartphone addiction in a Brazilian population of university students. Further studies should validate the SPAI-BR in other populations, such as children, elderly and clinical populations.

## Supporting information

S1 Fig Flow diagram and cross tabulation of the index test results by the results of the gold standard. (DOCX)

S2 Fig ROC curve of SPAI-BR. (DOCX)

S1 Table Original version and the Brazilian Portuguese adapted version of the SPAI. (DOCX)

S2 Table Spearman Correlation Coefficient between test and retest for the total SPAI-BR and the SPAI-BR’s factors (n = 130). Note: T1 = test of the factor “compulsive behavior”, T2 = test of the factor “functional impairment”, T3 = test of the factor “withdrawal”, T4 = test of the factor “tolerance”, TT = test of the total SPAI-BR, R1 = retest of the factor “compulsive behavior”, R2 = retest of the factor “functional impairment”, R3 = retest of the factor “withdrawal”, R4 = retest of the factor “tolerance”, RT = retest of the total SPAI-BR.
(DOCX)

S3 Table Psychometric values for SPAI-BR at each cut-off point taking into account different prevalences of smartphone addiction. Note: CI = confidence interval; +PV = Positive Predictive Value; -PV = Negative Predictive Value.
(DOCX)

S4 Table Relative costs associated with specific prevalences and optimal cut-off points. (DOCX)

S1 File Data bank. (SAV)
