# Supplementary material for: Assessment of the accuracy of a new tool for the screening of smartphone addiction
Source: PLoS One. 2017 May 17;12(5):e0176924. doi: 10.1371/journal.pone.0176924 (PMC5435144; doi:10.1371/journal.pone.0176924)
Supplement: S1 Fig — (DOCX) [file pone.0176924.s001.docx]

**S1 Fig. Flow diagram and cross tabulation of the index test results by the results of the gold standard.**
