# Supplementary material for: Assessment of the accuracy of a new tool for the screening of smartphone addiction
Source: PLoS One. 2017 May 17;12(5):e0176924. doi: 10.1371/journal.pone.0176924 (PMC5435144; doi:10.1371/journal.pone.0176924)
Supplement: S2 Fig — (DOCX) [file pone.0176924.s002.docx]

**S2 Fig. ROC curve of SPAI-BR**
