# Supplementary material for: Assessment of the accuracy of a new tool for the screening of smartphone addiction
Source: PLoS One. 2017 May 17;12(5):e0176924. doi: 10.1371/journal.pone.0176924 (PMC5435144; doi:10.1371/journal.pone.0176924)
Supplement: S1 Table — (DOCX) [file pone.0176924.s003.docx]

**S1 Table**: **Original version and the Brazilian Portuguese adapted version of the SPAI.**

| **SPAI original** | **SPAI-BR** |
| --- | --- |
| 1- I was told more than once that I spend too much time on the smartphone. | 1- Já me disseram mais de uma vez que eu passo tempo demais no smartphone. |
| 2- I feel uneasy once I stop using the smartphone for a certain period of time. | 2-Eu me sinto inquieto quando eu fico sem usar o smartphone durante um certo período de tempo. |
| 3- I think I have been hooking on smartphone longer and longer. | 3- Eu acho que eu tenho ficado cada vez mais tempo conectado ao smartphone. |
| 4- I feel restless and irritable when the smartphone is unavailable. | 4- Eu me sinto inquieto e irritado quando não tenho acesso ao smartphone. |
| 5- I feel very vigorous upon smartphone use regardless the fatigues experienced. | 5- Eu me sinto disposto a usar o smartphone mesmo quando me sinto cansado. |
| 6- I use the smartphone for a long period of time and spend more money than I have intended. | 6- Eu uso smartphone durante mais tempo e/ou gasto mais dinheiro nele do que eu pretendia inicialmente. |
| 7- Although using smartphone has brought negative effects on my interpersonal relationships, the amount of time spent on the Internet remains unreduced. | 7- Embora o uso de smartphone tenha trazido efeitos negativos nos meus relacionamentos interpessoais, a quantidade de tempo que eu gasto nele mantem-se a mesma. |
| 8- I have slept less than four hours due to using the smartphone more than once. | 8- Em mais de uma ocasião, eu dormi menos que quatro horas porque fiquei usando o smartphone. |
| 9- I have increased a substantial amount of time using smartphone per week in the last 3 months. | 9- Eu tenho aumentado consideravelmente o tempo gasto usando o smartphone nos últimos 3 meses. |
| 10- I feel distressed or down once I cease using the smartphone for a certain period of time. | 10- Eu me sinto incomodado ou para baixo quando eu paro de usar o smartphone por um certo período de tempo. |
| 11- I fail to control the impulse to use the smartphone. | 11- Eu não consigo controlar o impulso de utilizar o smartphone. |
| 12- I find myself indulged on the smartphone at the cost of hanging out with friends. | 12- Eu me sinto mais satisfeito utilizando o smartphone do que passando tempo com meus amigos. |
| 13- I feel aches and soreness in the back or eye discomforts due to excessive smartphone use. | 13- Eu sinto dores ou incômodos nas costas, ou desconforto nos olhos, devido ao uso excessivo do smartphone. |
| 14- The idea of using smartphone comes as the first thought on my mind when I wake up each morning. | 14- A ideia de utilizar o smartphone vem como primeiro pensamento na minha cabeça quando acordo de manha. |
| 15- The use of smartphone has exercised certain negative effects on my schoolwork or job performance. | 15- O uso de smartphone tem causado efeitos negativos no meu desempenho na escola ou no trabalho. |
| 16-I feel missing something after stopping smartphone for a certain period of time. | 16- Eu sinto falta de algo ao parar o uso do smartphone por um certo período de tempo. |
| 17- My interaction with family members has decreased on account of smartphone use. | 17- Minha interação com meus familiares diminuiu por causa do meu uso do smartphone. |
| 18-My recreational activities are reduced due to smartphone use. | 18- Minhas atividades de lazer diminuíram por causa do uso do smartphone. |
| 19- I feel the urge to use my smartphone again right after I stop using it. | 19- Eu sinto uma grande vontade de usar o smartphone novamente logo depois que eu paro de usa-lo. |
| 20- My life would be joyless hadn’t there been the smartphone. | 20- Minha vida seria sem graça se eu não tivesse o smartphone. |
| 21- Surfing the smartphone has exercised negative effects on my physical health. For example, viewing smartphone when crossing the street; fumbling with one’s smartphone while driving or waiting, and resulted in danger. | 21- Navegar no smartphone tem causado prejuízos para a minha saúde física. Por exemplo, uso o smartphone quando atravesso a rua, ou enquanto dirijo ou espero algo, e esse uso pode ter me colocado em perigo. |
| 22-I have tried to spend less time on the smartphone, but the efforts were in vain. | 22- Eu tenho tentado passar menos tempo usando o smartphone, mas não tenho conseguido. |
| 23- I make it a habit to use the smartphone and my sleep quality and total sleep time have decreased. | 23- Eu tornei o uso do smartphone um hábito e minha qualidade e tempo total de sono diminuíram. |
| 24- I need to spend an increasing amount of time on the smartphone to achieve the  same satisfaction as before. | 24- Eu preciso gastar cada vez mais tempo no smartphone para alcançar a mesma satisfação de antes. |
| 25- I can not have a meal without smartphone use. | 25- Eu não consigo fazer uma refeição sem utilizar o smartphone. |
| 26- I feel tired on daytime due to a late-night use of the smartphone. | 26- Eu me sinto cansado durante o dia devido ao uso do smartphone tarde da  noite/de madrugada. |
