# Supplementary material for: Assessment of the accuracy of a new tool for the screening of smartphone addiction
Source: PLoS One. 2017 May 17;12(5):e0176924. doi: 10.1371/journal.pone.0176924 (PMC5435144; doi:10.1371/journal.pone.0176924)
Supplement: S2 Table — Note: T1 = test of the factor “compulsive behavior”, T2 = test of the factor “functional impairment”, T3 = test of the factor “withdrawal”, T4 = test of the factor “tolerance”, TT = test of the total SPAI-BR, R1 = retest of the factor “compulsive behavior”, R2 = retest of the factor “functional impairment”, R3 = retest of the factor “withdrawal”, R4 = retest of the factor “tolerance”, RT = retest of the total SPAI-BR. (DOCX) [file pone.0176924.s004.docx]

**S2 Table: Intraclass-Correlation Coefficient between test and retest of SPAI-BR (n=130)**

|  |  | 95% CI | | F Test With True Value 0 | | | |
| --- | --- | --- | --- | --- | --- | --- | --- |
|  | ICC | Lower Bound | Upper Bound | Value | df1 | df2 | p |
| SPAI-BR | .926 | .894 | .948 | 27.552 | 129 | 129 | < .001 |

Note: CI= confidence interval; df=degree of freedom; ICC=Intraclass-Confidence Interval
