# Supplementary material for: Assessment of the accuracy of a new tool for the screening of smartphone addiction
Source: PLoS One. 2017 May 17;12(5):e0176924. doi: 10.1371/journal.pone.0176924 (PMC5435144; doi:10.1371/journal.pone.0176924)
Supplement: S3 Table — Note: CI = confidence interval; +PV = Positive Predictive Value; -PV = Negative Predictive Value. (DOCX) [file pone.0176924.s005.docx]

**S3 Table:** **Psychometric values for SPAI-BR at each cut-off point taking into account different prevalences of smartphone addiction**

**Note:** CI=confidence interval; +PV= Positive Predictive Value; -PV= Negative Predictive Value
