# Supplementary material for: Assessment of the accuracy of a new tool for the screening of smartphone addiction
Source: PLoS One. 2017 May 17;12(5):e0176924. doi: 10.1371/journal.pone.0176924 (PMC5435144; doi:10.1371/journal.pone.0176924)
Supplement: S4 Table — (DOCX) [file pone.0176924.s006.docx]

| **Criterion** | **Sensitivity %** | **Specificity %** | **Costs with 5% prevalence** | **Costs with 10% prevalence** | **Costs with 20% prevalence** | **Costs with 35.7% prevalence** | **Costs with 40% prevalence** |
| --- | --- | --- | --- | --- | --- | --- | --- |
| ≥0 | 100.0 | 0.0 | 0.0730 | 0.1160 | 0.2020 | 0.3370 | 0.3740 |
| ≥1 | 100.0 | 10.9 | 0.0700 | 0.1132 | 0.1995 | 0.3350 | 0.3721 |
| ≥2 | 99.3 | 21.0 | 0.0673 | 0.1106 | 0.1973 | 0.3334 | 0.3707 |
| ≥4 | 98.7 | 35.2 | 0.0634 | 0.1070 | 0.1957 | 0.3310 | 0.3685 |
| ≥3 | 98.7 | 28.5 | 0.0652 | 0.1087 | 0.1941 | 0.3322 | 0.3696 |
| ≥5 | 95.3 | 43.1 | 0.0614 | 0.1053 | 0.1930 | 0.3308 | 0.3686 |
| ≥6 | 92.6 | 50.2 | 0.0596 | 0.1037 | 0.1920 | 0.3306 | 0.3685 |
| ≥7 | 90.5 | 59.9 | 0.0570 | 0.1014 | 0.1902 | **0.3296** | **0.3677** |
| ≥8 | 84.5 | 67.8 | 0.0552 | 0.1000 | 0.1897 | 0.3305 | 0.3690 |
| ≥9 | 79.1 | 75.7 | 0.0533 | 0.0986 | 0.1891 | 0.3311 | 0.3701 |
| ≥10 | 73.7 | 82.4 | 0.0517 | 0.0974 | 0.1887 | 0.3320 | 0.3713 |
| ≥11 | 70.3 | 87.3 | 0.0506 | **0.0965** | **0.1883** | 0.3324 | 0.3719 |
| ≥12 | 60.8 | 90.6 | 0.0502 | 0.0967 | 0.1896 | 0.3355 | 0.3755 |
| ≥13 | 54.7 | 93.3 | 0.0498 | 0.0966 | 0.1903 | 0.3374 | 0.3777 |
| ≥14 | 45.3 | 95.5 | 0.0497 | 0.0971 | 0.1919 | 0.3407 | 0.3815 |
| ≥15 | 39.9 | 96.6 | **0.0497** | 0.0974 | 0.1928 | 0.3426 | 0.3836 |
| ≥16 | 32.4 | 97.8 | 0.0498 | 0.0979 | 0.1942 | 0.3453 | 0.3867 |
| ≥17 | 27.7 | 98.1 | 0.0499 | 0.0983 | 0.1951 | 0.3471 | 0.3887 |
| ≥18 | 20.3 | 99.3 | 0.0500 | 0.0989 | 0.1965 | 0.3498 | 0.3918 |
| ≥19 | 16.2 | 99.6 | 0.0502 | 0.0992 | 0.1973 | 0.3513 | 0.3935 |
| ≥20 | 10.8 | 99.6 | 0.0505 | 0.0998 | 0.1985 | 0.3535 | 0.3959 |
| ≥21 | 8.1 | 99.6 | 0.0506 | 0.1001 | 0.1991 | 0.3545 | 0.3971 |
| ≥22 | 6.8 | 99.6 | 0.0507 | 0.1003 | 0.1994 | 0.3551 | 0.3977 |
| ≥23 | 5.4 | 99.6 | 0.0508 | 0.1004 | 0.1997 | 0.3556 | 0.3983 |
| ≥24 | 2.7 | 99.6 | 0.0509 | 0.1007 | 0.2003 | 0.3567 | 0.3995 |
| ≥25 | 0.7 | 99.6 | 0.0510 | 0.1009 | 0.2007 | 0.3574 | 0.4004 |
| ≥26 | 0.0 | 100.0 | 0.0510 | 0.1009 | 0.2008 | 0.3576 | 0.4006 |
